# From technology anxiety to technology symbiosis: a grounded theory study of mid-career foreign language teachers’ subjectivity evolution in the AI era

**DOI:** 10.3389/fpsyg.2026.1914637

**Published:** 2026-09-17

**Authors:** Zhenhua Zhao, Dengbin Xie

**Affiliations:** 1 Education Department, Guangxi Normal University, Guilin, China; 2 School of Foreign Languages and International Trade, Guilin University of Aerospace Technology, Guilin, China

**Keywords:** foreign language teachers, grounded theory, mid-career teachers, technology anxiety, technology symbiosis

## Abstract

The integration of generative AI into foreign language education has profoundly challenged teacher professional identity, particularly for mid-career educators navigating the dual burden of experiential wisdom and technological change. This study employed grounded theory to investigate how 10 mid-career foreign language teachers transition from technology anxiety to symbiosis. Through in-depth interviews and systematic coding, we developed a theoretical model comprising four anxiety types (substitution, autonomy, generational, ethical), three adaptive stages (exploration adjustment, strategy integration, autonomous symbiosis), and the double-edged effects of experiential wisdom as the core mediating mechanism. Findings reveal that experiential wisdom can function as either an evolutionary resource (critical scrutiny, pedagogical design, humanistic commitment) or a barrier (thinking set inertia) depending on its activation mode, with support systems moderating this process. The study reveals the existential dimensions of technology anxiety and offers stage-sensitive implications for supporting mid-career teachers’ professional growth in the AI era.

## 1 Introduction

The rapid integration of generative artificial intelligence (AI) into education is fundamentally reshaping language teaching, challenging not only pedagogical practices but the very foundations of teachers’ professional authority and identity (Selwyn, 2022). Within this transformative landscape, empirical studies have documented that “teachers’ level of technological anxiety has reached a relatively high level”, with “significant differences in AI anxiety across age groups” (Zhao et al., 2022). Mid-career teachers, particularly those aged 40 and above, have attracted considerable research attention due to their heightened vulnerability to technology-induced stress. This group occupies a unique intersection between the “career plateau” which is a stage characterized by stabilized professional routines and diminished promotion prospects (Ismail, 2008), and the “digital generational divide,” which amplifies their sense of displacement in technology-mediated environments. Unlike digital natives (their students and younger colleagues), mid-career teachers must navigate the dual burden of accumulated experiential wisdom and the demand for continuous technological upskilling. This convergence renders them particularly susceptible to professional disorientation and the erosion of their subjective agency. When AI demonstrates superior efficiency in tasks once reserved for human experts, such as translation, error correction, even conversational practice their professional identity is no longer unquestioned, prompting what Nguyen et al. (2025) term an “existential crisis” of teacher subjectivity.

Despite growing scholarly attention to teacher-technology relationships, existing research remains constrained in three respects. First, much of the literature adopts a technocentric perspective, focusing predominantly on technology acceptance models (TAM) or skill-based training interventions (Chen and Chen, 2025; Wang and Wang, 2019), treating teachers as homogeneous actors. While models such as TPACK (Koehler and Mishra, 2009) and the Concerns-Based Adoption Model (Hall and Hord, 2015) have advanced understanding of the knowledge and concerns involved in technology integration, they focus primarily on the technology itself and do not address how teachers’ accumulated professional experience mediates their adaptation leaving the identity-laden processes of navigating technological change largely untheorized.

Second, although scholars have increasingly recognized that AI anxiety extends beyond psychological discomfort to encompass existential concerns about role obsolescence and value erosion (Wang and Wang, 2024; Zhao et al., 2024), few studies have theorized the mechanisms through which teachers might transcend anxiety toward a state of productive symbiosis with technology. The concept of “technology symbiosis” has its conceptual origins in Licklider’s (1960) classic formulation of “man-computer symbiosis,” which he defined as a cooperative interaction between men and electronic computers involving “very close coupling between the human and the electronic members of the partnership”. In this vision, humans and computers form a symbiotic partnership in which men will set the goals, formulate the hypotheses, determine the criteria, and perform the evaluations, while computing machines will “do the routinizable work”. Contemporary international scholarship has extended this concept to educational contexts, examining how AI systems function not merely as tools but as “active agents” that reshape teachers’ pedagogical approaches (Liu and Qin, 2025). Recent studies have documented how teachers experience a shift from an initial crisis of authority to a reconstructed identity as AI collaborators, while posthumanist perspectives have reframed AI as a “co-creative presence” that invites new forms of human-machine collaboration in pedagogy. The concept of “technology symbiosis” characterized by dynamic equilibrium, mutual adaptation, and value co-creation between human agents and technological systems has been proposed as a desirable endpoint (Fang, 2025; Du and Ruan, 2025), yet existing research has primarily conceptualized it at the systems level, without specifying how individual teachers achieve such symbiosis through identity reconstruction.

Third, and most critically, the specific experiences of mid-career foreign language teachers have been largely neglected in both technology anxiety research and teacher professional development scholarship. This omission is problematic because this group embodies a distinctive configuration of resources and constraints: their extensive pedagogical content knowledge positions them as potential innovators, yet their entrenched routines may impede adaptation (Sun and Tian, 2020). Understanding how experiential wisdom functions as both asset and barrier is essential for designing targeted interventions.

Notably, the Chinese higher education context provides a theoretically important setting for investigating this phenomenon for three interrelated reasons. First, China’s national-level AI-in-education policies (Ministry of Education of China, 2018, 2024) create institutional conditions where technology adoption is strongly mandated, intensifying autonomy anxiety when teachers feel their pedagogical agency is constrained. Second, seniority-based norms in Chinese academia extend generational anxiety, when mid-career teachers who are respected for their experience, finding themselves less technologically fluent than junior colleagues and students (Zhao et al., 2022). Third, the rapid proliferation of generative AI tools specifically designed for English language education in China (e.g., intelligent essay scoring, AI-powered oral assessment) provides a concentrated and visible site of technological disruption, making foreign language teachers’ adaptation experiences both salient and empirically accessible. These contextual features render China a critical case for examining mid-career teacher subjectivity evolution.

To address these problems, this study adopts a constructivist grounded theory approach to investigate how mid-career foreign language teachers in Chinese universities navigate the transition from technology anxiety to technology symbiosis. Drawing upon Heidegger’s (1962) existential phenomenology and dynamic theories of professional identity construction (Day, 2011), we conceptualize teacher subjectivity not as a static essence but as an emergent, relational achievement shaped through ongoing interactions with technology, students, and institutional contexts. Three research questions guide the inquiry:

1 What are the multidimensional manifestations and core sources of technology anxiety experienced by mid-career foreign language teachers?
2 Through what phased adaptive pathways does their subjectivity evolve from anxiety toward symbiosis?
3 What key factors drive or moderate this adaptive process?

By elucidating the mechanisms underlying mid-career teachers’ subjective reconstruction, this study makes three contributions. Theoretically, it challenges deficit-oriented narratives by revealing experiential wisdom’s double-edged role. Empirically, it offers grounded theory of this population’s adaptation pathways. Practically, it offers actionable guidance for designing differentiated support systems that cultivate teachers’ potential as humanistic leaders in AI-enriched environments.

## 2 Theoretical framework and literature review

### 2.1 Teacher subjectivity: theoretical foundations and contemporary challenges

The concept of subjectivity has deep philosophical roots that inform contemporary understandings of teacher professional identity and agency. In philosophy, especially the Enlightenment tradition, particularly Descartes’ “Cogito, ergo sum” (I think, therefore I am), established the thinking subject as the foundation of knowledge and certainty (Descartes, 1961/1984). Kant further consolidated this anthropocentric orientation by arguing that objects conform to human cognition rather than the reverse, thereby positioning the human subject at the center of epistemological relations (Kant, 2003). While these foundational perspectives established the autonomous, rational subject as the locus of agency, they also implicitly framed the relationship between human and technology in dualistic terms that becomes increasingly apparent in contemporary technology-saturated educational environments.

Heidegger’s existential phenomenology offers a more productive lens for understanding teacher-technology relations. His concepts of Dasein (being-there) and Mitsein (being-with) reconceptualize human existence as fundamentally situated and relational (Heidegger, 1962). From this perspective, teachers do not simply “use” technology as an external tool; rather, their professional being is constituted through engagement with technological artifacts, colleagues, students, and institutional structures. This ontological turn suggests that teacher subjectivity is not a pre-existing essence that technology subsequently “impacts,” but rather an emergent property of situated practice within specific sociomaterial configurations.

In traditional educational contexts, teacher subjectivity has been operationalized through two interrelated dimensions: pedagogical authority and curriculum agency. Shulman (1987) famously articulated the knowledge base for teaching, positioning teachers as authoritative decision-makers who integrate content knowledge, pedagogical knowledge, and contextual knowledge in complex practice. Similarly, Connelly and Clandinin (1988) emphasized teachers’ role as curriculum makers, arguing that curriculum is not merely implemented but actively created through teachers’ narrative experiences and practical knowledge. These perspectives established what might be termed the “teacher-as-professional-authority” paradigm, wherein teacher subjectivity is expressed through autonomous judgment, creative adaptation, and principled action within classrooms.

Concurrently, teacher identity research has highlighted the ongoing, dynamic nature of professional self-understanding. Identity is not a fixed attribute but an ongoing interpretive process which answers to the question “who am I as a teacher?”, which are constructed through interaction with social, cultural, and institutional environments (Day, 2011; Rodgers and Scott, 2008). Teachers’ professional identities are shaped by personal biographies, workplace conditions, policy contexts, and relationships with students and colleagues, and they remain subject to continuous negotiation and reconstruction.

However, the integration of generative AI into foreign language education fundamentally disrupts these established foundations of teacher subjectivity. Several interrelated challenges emerge from this technological transformation:

First, the evolution of AI from tool to quasi-agent problematizes the human-technology boundary (Liu and Qin, 2025). When AI systems independently generate content, provide feedback, and adapt learning pathways, teachers can no longer maintain the subject-object dichotomy that positioned them as sovereign users of inert instruments. Instead, they confront an entity that acts with them and sometimes in ways that challenge their pedagogical judgments.

Second, AI demonstrated capabilities in domains traditionally reserved for language teachers, such as translation, text generation, error analysis, even conversational practice, which undermine teachers’ epistemological authority. If an AI can produce grammatically correct texts, identify language errors, and engage students in meaningful dialogue, what distinctive expertise do human teachers possess? This question strikes at the heart of teachers’ professional identity and self-worth.

Third, the standardization inherent in algorithmic pedagogy threatens teachers’ creative autonomy. AI-recommended content, platform-mandated workflows, and pre-designed activity templates can constrain teachers’ capacity for improvisation, emotional attunement, and context-sensitive adaptation (Wang and Wang, 2024). As Popenici and Kerr (2017) caution, when teaching becomes algorithmically mediated, teachers risk being reduced to “data appendages” whose judgments are subordinate to computational prescriptions.

Fourth, the relational fabric of teaching is reconfigured when students increasingly interact with AI systems. Language learning, fundamentally a humanistic endeavor centered on communication and cultural understanding, becomes partially mediated by non-human agents, potentially attenuating the teacher-student bond that constitutes the affective core of education (Zhao et al., 2024).

These challenges expose the limitations of existing conceptualizations of teacher subjectivity. Much of the literature remains trapped within subject-object dualism, treating technology as an external force that impacts pre-constituted subjects (Biesta, 2020). Alternatively, some approaches reify teacher subjectivity as a stable essence to be protected against technological encroachment. Neither perspective adequately captures the dynamic, co-constitutive relationship between teachers and technologies in AI-enriched environments.

The concept of “humanistic leaders” in this paper refers to a reconstructed professional identity in the AI era, in which teachers consciously anchor their irreplaceable value in distinctively human educational capacities: fostering emotional connection, providing value guidance, cultivating independent critical thinking, and attending to the holistic development of each student (Biesta, 2017; Zhao et al., 2024). Unlike AI systems, which excel at information processing and pattern recognition, humanistic leaders foreground the relational and ethical dimensions of education that remain beyond technological replication. This conceptualization serves as the normative endpoint of the subjectivity evolution pathway we investigate.

Therefore, this study advances an integrated theoretical framework that conceptualizes mid-career foreign language teachers’ subjectivity evolution through two complementary lenses: (1) existential subjectivity, which understands teachers’ relationship with technology as a mode of professional “being-in-the-world” (Heidegger, 1962); (2) dynamic identity construction, which views subjectivity evolution as teachers’ ongoing effort to reconstruct professional identity amid internal and external adaptation demands (Day, 2011). This framework moves beyond protectionist or defensive stances, instead recognizing that teacher subjectivity is not merely preserved but potentially transformed and enriched through productive engagement with technology.

### 2.2 Teacher technology anxiety: from psychological construct to existential crisis

The concept of technology anxiety originated in human-computer interaction research, referring to individuals’ affective and behavioral responses, such as fear, apprehension, and avoidance, when confronted with technology (Marcoulides and Wang, 1990). Early research predominantly followed the “stimulus–response” paradigm or employed Technology Acceptance Models (TAM) to investigate how perceived ease of use and usefulness influenced technology adoption (Davis, 1989). Within this tradition, anxiety was conceptualized as a barrier to acceptance, and skill deficits were identified as its primary cause, leading to recommendations for training interventions (Wang and Wang, 2019; Chen and Chen, 2025).

As artificial intelligence technologies have advanced, scholarship has progressively differentiated general “technology anxiety” from more specific “AI anxiety.” Contemporary researchers conceptualize AI anxiety as a multidimensional negative emotional complex encompassing affective, cognitive, and existential dimensions (Wang et al., 2022). Unlike anxiety about conventional digital tools, AI anxiety is characterized by concerns about job displacement, loss of human control, and fundamental challenges to professional identity and value. As Selwyn (2022) argues, AI anxiety’s deep structure reflects technology’s capacity to disrupt educational essence, teacher roles, and human relationships-threatening the very foundations of teachers’ subjective agency.

Recent scholarship has thus shifted from psychological toward sociological and philosophical perspectives. Rather than asking merely “how can we reduce anxiety?”, researchers increasingly inquire: “what does this anxiety reveal about teachers’ existential situation in technologically saturated environments?” (Zhao et al., 2024). This reframing recognizes anxiety not simply as a problem to be eliminated, but as an existential signal that teachers’ professional being is at stake, demanding new modes of self-understanding and practice (Biesta, 2020). Studies have documented that teachers’ AI anxiety encompasses concerns about professional replacement, pedagogical autonomy, ethical implications, and intergenerational competition (Zhao et al., 2022). Importantly, researchers have increasingly recognized that resolving AI anxiety requires not merely skill training but the cultivation of new forms of technology-teacher symbiosis (Fang, 2025; Du and Ruan, 2025).

In this study, we adopt the term “technology symbiosis” to describe a qualitatively distinct state in teachers’ relationship with AI technology. However, we wish to clarify that our usage carries an analytical scope that differs from the classic systems-level definition. As employed in our grounded theory model, technology symbiosis refers to a state of teacher-perceived harmonious collaboration in which teachers have reconstructed their professional subjectivity to the extent that technology is experienced as an empowering partner rather than a threatening force. This definition is intentionally teacher-centric: it captures how teachers experience, perceive, and narrate their relationship with technology. Through sustained interaction and mutual adaptation, teachers and technologies achieve a state of dynamic equilibrium wherein technology enhances rather than diminishes human agency (Du and Ruan, 2025). The essence of symbiosis is teachers’ reaffirmation and elevation of their professional subjectivity as “humanistic leaders” and “learning designers” that AI cannot replicate. Thus, the trajectory “from technology anxiety to technology symbiosis” constitutes the core pathway of teacher subjectivity evolution in the digital age.

This study focuses on mid-career foreign language teachers in higher education. Employing grounded theory methodology, we investigate how mid-aged population navigates the transition from technology anxiety to technology symbiosis, attending to within-group variation and the mechanisms through which experiential wisdom functions as both resource and barrier in this adaptive journey.

## 3 Methodology

### 3.1 Research design

This study employed a constructivist grounded theory approach (Charmaz, 2014), which is particularly suited for exploring the dynamic, under-theorized process of transitioning from technology anxiety to technology symbiosis among mid-career foreign language teachers. The research design proceeded through three integrated phases:

1 An initial questionnaire survey to map the broad contours of technology anxiety within the target population and to identify potential participants for in-depth inquiry.
2 Semi-structured interviews with a purposively selected sample maximizing variation on key dimensions.
3 Systematic grounded theory analysis involving open, axial, and selective coding to develop a theoretical model of subjectivity evolution. This phased approach enabled both breadth in capturing population characteristics and depth in elucidating underlying mechanisms.

### 3.2 Sampling and participants

#### 3.2.1 Initial screening survey

The study targeted in-service university foreign language teachers aged 40 years and above in Chinese higher education institutions. An initial screening questionnaire was distributed through two primary channels: recommendations from foreign language department administrators at partner universities, and targeted distribution through professional teacher communities on social media platforms (WeChat and QQ groups). The questionnaire assessed demographic characteristics (age, years of teaching experience, academic rank) and included items probing teachers’ self-perceived technology anxiety, technology competence (self-rated on a 5-point scale: 1 = very low, 5 = very high), and openness to technology integration.

A total of 114 valid responses were received, yielding an effective response rate of 95.3%. The questionnaire demonstrated satisfactory reliability and validity (KMO = 0.791; Cronbach’s α = 0.872 for the full scale). It is important to note that the questionnaire served a screening and contextualization function rather than hypothesis testing; its primary purpose was to enable theoretically grounded sampling for the qualitative phase (Morse, 2007).

#### 3.2.2 Participant selection for in-depth interviews

Following the principle of maximum variation sampling (Patton, 2015), we purposefully selected 10 participants from the initial respondent pool to ensure heterogeneity across theoretically relevant dimensions: age, years of teaching experience, academic rank (lecturer, associate professor, professor), institutional type (comprehensive university, science and technology university, normal university, foreign studies university), self-rated technology competence, and self-reported anxiety level. This sampling strategy aimed to capture the full spectrum of possible adaptation pathways within the mid-career teacher population, thereby enhancing the theoretical reach of the emergent model (Corbin and Strauss, 2008).

Sample size was determined by the principle of theoretical saturation at which additional data collection no longer yields new conceptual insights or modifies emerging theoretical categories (Glaser and Strauss, 1967). After completing interviews with the 10th participant and conducting concurrent coding, core categories had stabilized and no new themes emerged, indicating that saturation had been achieved.

Table 1 presents the demographic characteristics of the 10 interview participants. To protect anonymity, each participant is identified by a code (N1–N10).

**Table 1:** Participant demographic characteristics.

Code	Institutional type	Teaching experience (years)	Age	Academic rank	Self-rated technology competence (1–5)
N1	Science and Technology Univ.	18	44	Lecturer	4
N2	Comprehensive University	23	48	Associate Prof.	2
N3	Science and Technology Univ.	17	43	Lecturer	3
N4	Comprehensive University	17	43	Professor	3
N5	Comprehensive University	27	50	Associate Prof.	3
N6	Normal University	18	42	Associate Prof.	2
N7	Science and Technology Univ.	24	48	Professor	3
N8	Science and Technology Univ.	22	45	Associate Prof.	3
N9	Science and Technology Univ.	29	59	Associate Prof.	4
N10	Foreign Studies University	19	44	Associate Prof.	3

Table presents the demographic characteristics of the 10 interview participants. To protect anonymity, each participant is identified by a code (N1-N10). One participant (N9, aged 59) may be more accurately characterized as late-career rather than mid-career. We have therefore referred to our population as “mid- to late-career teachers” where appropriate throughout the manuscript. In the Chinese higher education context, under the National Silver Age Teacher Initiative and flexible retirement policies, faculty with senior professional titles may have extended working lives, meaning a 59-year-old male with a senior title may not self-identify as “late-career” in the traditional sense.

### 3.3 Data collection

#### 3.3.1 Semi-structured interview protocol

Data were collected through in-depth, semi-structured interviews conducted between May and July 2025. Each interview lasted approximately 40–60 min and was guided by a flexible protocol designed to explore participants’ experiences, perceptions, and meaning-making processes regarding AI technology integration. The protocol encompassed five thematic modules and is presented in Appendix B.

Professional identity and self-understanding: How do participants perceive their roles and professional selves in the context of AI integration? (e.g., “How would you describe your professional identity as a language teacher today, compared to five years ago?”)

Technology interaction experiences: What are participants’ actual experiences with AI tools in teaching? (e.g., “Can you describe a specific situation where you used AI in your teaching? What happened?”)

Affective responses and anxiety manifestations: How do participants experience and make sense of technology-related anxiety? (e.g., “What aspects of AI technology, if any, cause you concern or unease? Can you give an example?”)

Role perceptions and boundary negotiations: How do participants perceive the changing boundaries between teacher and technology? (e.g., “In your view, what should teachers do that AI cannot or should not do?”)

Support systems and adaptive strategies: What resources and strategies do participants mobilize in adapting to AI? (e.g., “What or who has been most helpful in your process of learning to work with AI?”)

The protocol was pilot-tested with two mid-career teachers not included in the final sample, leading to minor refinements in question wording and sequencing to enhance clarity and relevance.

#### 3.3.2 Interview procedures

Interviews were conducted in Mandarin Chinese, the participants’ and first author’s native language, to enable authentic expression without linguistic constraint. All research instruments were developed and administered in Chinese; data were subsequently translated into English for manuscript preparation. Interview quotations were translated by the first author, a bilingual researcher, with attention to preserving both semantic accuracy and cultural nuance. Participants chose the interview setting (online via Tencent Meeting or in-person at their university offices) based on convenience. All interviews were audio-recorded with participants’ informed consent and transcribed verbatim immediately afterward. Transcripts were checked for accuracy by comparing them with the audio recordings. To ensure confidentiality, all identifying information (names, institutions, specific course details) was removed or pseudonymized during transcription. Field notes documenting contextual observations and reflexive memos capturing emerging analytical insights were maintained throughout the data collection period (Charmaz, 2014).

### 3.4 Data analysis

Data analysis followed grounded theory procedures of open, axial, and selective coding (Corbin and Strauss, 2008), conducted iteratively and concurrently with data collection. NVivo 12 software was used to manage and code data.

#### 3.4.1 Open coding

Open coding involved line-by-line examination of interview transcripts to identify discrete concepts embedded in participants’ accounts. Through constant comparison, semantically similar codes were merged, yielding 25 preliminary concepts. Axial coding synthesized these open codes into higher-level categories and specified relationships among them. To enhance analytical rigor, a dual-coding procedure was implemented. Two researchers independently coded the first three interview transcripts. Inter-coder reliability was calculated using Cohen’s Kappa, yielding κ = 0.84, indicating strong agreement (Landis and Koch, 1977). Discrepancies, which primarily arose from differences in the level of abstraction, were resolved through consensus discussion with reference to the original data. After achieving consensus, the first author coded the remaining transcripts, with the research assistant independently coding 20% of additional transcripts to ensure ongoing reliability (κ = 0.81 for the second round). The complete set of 25 initial concepts is presented in Appendix A. Table 2 presents the resulting core categories with representative illustrative quotations.

**Table 2:** Examples of open coding.

Conceptual label	Illustrative quotation (participant code)
Technological shock	“I found it incredibly powerful. At that moment, I was very anxious. I wondered whether we still needed English majors or translation majors. The pressure was immense.” (N1)
Pedagogical pain points	“My university English classes have very large enrollments. the enormous workload causes serious teaching difficulties.” (N10)
Exploration-stage characteristics	“If you are asking about AI technology and platforms, I’d rate myself 2 points—I’m still a beginner, only using basic functions.”(N2)
Integration-stage relational anxiety	“This is my biggest anxiety as a teacher… discovering that students’ AI-written content constitutes a high proportion of their submissions.” (N5)
Experience as resource: Critical scrutiny	“Relying on our independent thinking abilities and long-term disciplinary accumulation, we can identify problematic stances and angles in AI-generated content.” (N9)
Experience as barrier: Thinking set	“The teaching methods, styles, classroom pacing, and interaction patterns formed over many years are relatively rigid.” (N2)
Community: Teacher-student co-learning	“I explore together with my students… students comprehend faster than I do, and after they learn, they teach me.” (N5)
Training: Need for precision	“I prefer targeted training lasting about a week… for older teachers, a model combining training with practice works better.” (N7)
Task design innovation	“I design more complex tasks… requiring students to consult materials and read extensively to solve problems.” (N1)
Adherence to humanistic values	“Taking essay grading as an example, AI has deficiencies in emotional judgment. For the same essay, from the perspective of emotional expression, a teacher’s score may be more than 10 points higher than AI’s.” (N-7)

#### 3.4.2 Axial coding

Axial coding aimed to synthesize open codes into higher-level categories and specify the relationships among them (Corbin and Strauss, 2008). This phase involved identifying the central phenomenon (technology anxiety as a multifaceted trigger), causal conditions (the four anxiety types), contextual factors (career stage, institutional environment), intervening conditions (experiential wisdom’s dual effects), action strategies (adaptive behaviors), and consequences (evolutionary stages).

Table 3 presents the axial coding structure, illustrating how lower-level concepts were organized under these core categories.

**Table 3:** Axial coding structure: Core categories, subcategories, and conceptual definitions.

Core category	Subcategories	Conceptual definition
	Ethical anxiety	Concern about academic integrity breakdown and transformed student learning behaviors due to AI misuse
	Autonomy anxiety	Resistance and distress when institutional policies mandate or strongly prescribe specific AI uses, constraining pedagogical agency
	Generational anxiety	Identity threat arising from perceived gaps in technological fluency compared with younger colleagues and students
Adaptive states	Exploration-adjustment characteristics	Initial attempts to use technology, with focus on basic skill acquisition
	Strategy-integration characteristics	Conscious design of pedagogical strategies to address technology-induced challenges, with attention to shifting teacher-student relationships
	Autonomous-symbiosis characteristics	Agentic mastery of technology, characterized by harmonious collaboration and reaffirmed professional core values
Experiential wisdom’s dual effects	Critical scrutiny capacity	Ability to evaluate AI outputs based on accumulated disciplinary knowledge
	Pedagogical design wisdom	Capacity to creatively integrate technology into established teaching frameworks
	Humanistic value commitment	Deliberate emphasis on affective interaction, thinking cultivation, and value guidance
	Thinking set inertia	Resistance to change rooted in entrenched teaching routines
Support systems	Collegial networks	Formal and informal peer support for technology learning
	Teacher-student learning communities	Reciprocal learning arrangements where teachers and students explore AI together
	Training precision needs	Expectations for differentiated, practice-oriented professional development
	Resource adequacy needs	Requirements for equipment, funding, and protected time
Autonomous symbiosis	Critical integration	Selective adoption and modification of AI outputs based on professional judgment
	Task design innovation	Creating complex tasks that require genuine student engagement beyond AI generation
	Role redefinition	Recalibrating boundaries between teacher and technology responsibilities
	Agentic learning exploration	Active technology mastery through self-study, consultation, and training

#### 3.4.3 Selective coding

The final phase, selective coding, involved integrating the core categories into a coherent theoretical framework that explains the central phenomenon of subjectivity evolution (Corbin and Strauss, 2008). Through constant comparison and theoretical memoing, we identified “mid-career foreign language teachers’ subjectivity evolution mechanism” as the core category which all other categories were related.

The core category represents a processual model wherein technology anxiety serves as the initial driver, experiential wisdom’s dual effects function as the pivotal mediating mechanism, and support systems moderate the relationship between anxiety and adaptive outcomes. Teachers, through their action strategies, progress through three adaptive states (exploration-adjustment → strategy integration → autonomous symbiosis), ultimately achieving a reconstructed professional subjectivity characterized by harmonious human-technology coexistence.

To enhance theoretical validity, we subjected the emerging model to member checking (Lincoln and Guba, 1985): three participants reviewed the theoretical framework and confirmed that it resonated with their experiences. Additionally, peer debriefing with two researchers familiar with teacher development and technology integration provided critical feedback that refined category definitions and relational claims.

#### 3.4.4 Trustworthiness and rigor

Following Lincoln and Guba’s (1985) criteria for naturalistic inquiry, we implemented multiple strategies to ensure trustworthiness:

1 Credibility: Prolonged engagement with the phenomenon (the first author is a mid-career foreign language teacher), triangulation across data sources (questionnaire, interviews, field notes), member checks, and peer debriefing enhanced the likelihood that findings credibly represent participants’ experiences.
2 Transferability: Thick description of participants, contexts, and processes enables readers to assess the applicability of findings to other settings. Maximum variation sampling enhanced the theoretical scope of the emergent model.
3 Dependability and confirmability: An audit trail documenting sampling decisions, interview protocols, coding schemes, analytical memos, and theoretical refinements was maintained throughout the research process. All analytical claims can be traced back to data sources, ensuring that interpretations are grounded in evidence rather than researcher preconceptions.

### 3.5 Ethical considerations

All participants were informed of the research purposes, procedures, and their rights to withdraw at any time without consequence. To protect confidentiality, all identifying information has been removed from this manuscript, and pseudonyms are used in all reporting. Participants were offered the opportunity to review their interview transcripts and request deletions or modifications, though none chose to do so.

## 4 Findings

This section presents the theoretical model of subjectivity evolution that emerged from the grounded theory analysis. We first introduce the overall framework, then elaborate each core category and its interrelationships, illustrated with representative quotations from participants. The model, depicted in Figure 1, captures the dynamic process through which mid-career foreign language teachers navigate the transition from technology anxiety to technology symbiosis.

**Figure 1:**
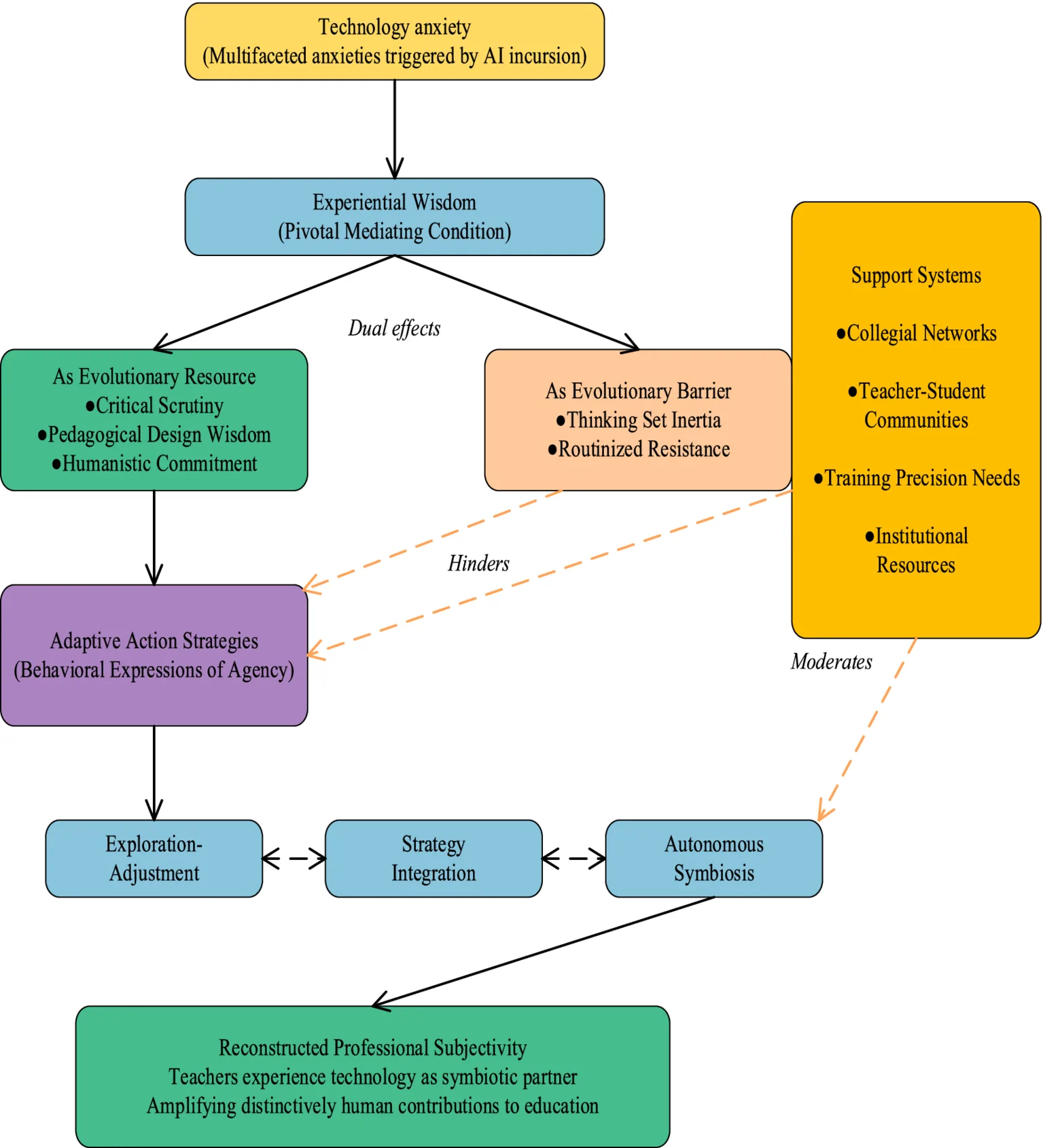
Mid-career foreign language teachers’ subjectivity evolution mechanism. Solid arrows indicate relationships consistently supported across all 10 participants’ accounts. Dashed arrows indicate conditional relationships that emerged only under specific activation modes of experiential wisdom or in particular institutional contexts. The bidirectional arrows among the three adaptive orientations signify that progression is non-linear and iterative, since teachers may oscillate between orientations or regress to earlier ones depending on how their experiential wisdom is mobilized and the support available.

The theoretical model depicted in Figure 1 integrates five components: technology anxiety as trigger, experiential wisdom as mediating mechanism, support systems as moderating factors, action strategies as behavioral expressions, and adaptive orientations as outcomes. Solid arrows indicate direct causal relationships consistently identified across all participants’ accounts. Dashed arrows indicate conditional relationships that emerged only under specific institutional contexts or activation modes. Double-headed arrows among the three orientations signify non-linear progression since teachers may oscillate between or regress among orientations depending on how their experiential wisdom is mobilized and the support available.

### 4.1 The subjectivity evolution mechanism: an overview

The core category identified through selective coding that is the mid-career foreign language teachers’ subjectivity evolution mechanism, integrating five major components: (1) technology anxiety as the initial trigger, (2) experiential wisdom’s dual effects as the pivotal mediating condition, (3) support systems as moderating factors, (4) adaptive action strategies as the behavioral expressions of agency, and (5) progressive adaptive states as the developmental outcomes. As illustrated in Figure 1, the process unfolds as follows:

As illustrated in Figure 1, the process unfolds as follows. Driven by multifaceted anxieties triggered by AI incursion into language teaching, teachers activate their accumulated experiential wisdom. This activation is causal and consistently observed across all participants, all of whom described anxiety as the initial impetus that prompted them to draw upon their pedagogical experience. Depending on how this wisdom is mobilized, whether as an evolutionary resource (critical scrutiny, pedagogical creativity, humanistic commitment) or as an evolutionary barrier (thinking set inertia). Teachers engage in qualitatively different action strategies. The relationship between wisdom activation and strategy selection is conditional. The same experiential repertoire can lead to resource-driven innovation or barrier-driven resistance depending on contextual triggers and the teacher’s reflexive orientation toward change. These strategies, in turn, shape their progression through three adaptive orientations: exploration-adjustment, strategy integration, and autonomous symbiosis. Notably, progression through these orientations is non-linear and bidirectional since teachers may move forward, stagnate, or regress between orientations. Throughout this trajectory, support systems (collegial networks, teacher-student communities, training, institutional resources) moderate the ease and effectiveness of adaptation. The outcome is a reconstructed professional subjectivity wherein teachers no longer experience technology as a threat but as a symbiotic partner that amplifies their distinctively human contributions to education.

### 4.2 Multifaceted technology anxiety: the trigger of adaptation

Technology anxiety among mid-career foreign language teachers is not a monolithic phenomenon. Analysis revealed four distinct yet interrelated types of anxiety, each originating from different aspects of AI encroachment into professional practice. These anxieties collectively constitute the emotional and existential impetus that propels teachers toward adaptive action.

#### 4.2.1 Substitution anxiety

Substitution anxiety refers to the profound existential unease triggered by AI demonstrated capacity to perform tasks that teachers have long considered the core of their professional expertise, such as language production, translation, error correction, and even instructional explanation. This anxiety strikes at the heart of professional identity, raising fundamental questions about the continued relevance of language teachers in an AI-saturated world.

“When I first encountered ChatGPT, I found it incredibly powerful. At that moment, I was very anxious. I wondered whether we still needed English majors or translation majors. The pressure was immense.” (N1, Lecturer, 44 years).

“I compared AI translations with human translations. AI translations were both faster and more accurate. It was a real shock—if machines can do this, what is left for us?” (N5, Associate Professor, 50 years).

Substitution anxiety is particularly acute among mid-career teachers because their professional identities have been forged over decades of mastering precisely the skills that AI now seemingly renders obsolete. The efficiency and quality of AI outputs confront them with an uncomfortable possibility: that their hard-won expertise may be devalued or displaced.

#### 4.2.2 Autonomy anxiety

Autonomy anxiety arises when institutional policies, management systems, or evaluation criteria mandate or strongly prescribe specific uses of AI, thereby constraining teachers’ pedagogical agency. Unlike substitution anxiety, which concerns the content of professional work, autonomy anxiety concerns the control over professional decision-making.

“Some university management systems force to use AI functions… I can only cope with such a mandatory requirement.” (N9, Associate Professor, 59 years).

“Take the Chaoxing platform as an example… initially teachers were very resistant because it increases without clear pedagogical benefits. We felt we were being forced into a mold.” (N4, Professor, 43 years).

Under conditions of compulsory technology integration, teachers experience a shift from “active designers” to “passive executors.” Their accumulated pedagogical wisdom what works for their specific students, in their specific contexts subordinated to standardized technological prescriptions. This erosion of professional autonomy triggers resistance and distress, yet also motivates some teachers to seek ways to reclaim agency within technologically constrained environments.

#### 4.2.3 Generational anxiety

Generational anxiety emerges from the perceived digital divide between mid-career teachers and their younger counterparts, both students and junior colleagues. This anxiety is not merely about skill deficits but about the deeper implications of these gaps for professional relevance and status.

“Our anxiety has two aspects: first, it is the lower acceptance of new technology. Initially we resist, feeling it’s unnecessary to learn; second, it is the slower learning speed that we forget quickly if we don’t practice immediately. In both respects, we lag behind younger teachers and students.” (N3, Lecturer, 43 years).

“In AI applications, we are definitely not as proficient as younger teachers. We know less about the characteristics of different AI tools and are less skilled when using them for teaching support. It takes us more time to explore.” (N7, Professor, 48 years).

Generational anxiety is intensified by the cultural context of Chinese higher education, where respect for seniority traditionally confers authority. When senior teachers find themselves less technologically fluent than those they are expected to lead or mentor, their professional identity suffers. Paradoxically, this anxiety can also become a motivational force, spurring mid-career teachers to “catch up” to preserve their standing.

#### 4.2.4 Ethical anxiety

Ethical anxiety stems from the disruption of academic integrity and traditional teaching-learning relationships caused by students’ widespread use of generative AI. It concerns about academic integrity breakdown and transformed student learning behaviors due to AI misuse. Teachers confront a transformed classroom reality wherein assignments that once demonstrated learning can now be produced with minimal student effort.

“In role-play tasks, students now directly input the task into AI, have it generate the dialogue, and then simply copy it. There’s no thinking process involved anymore.” (N1, Lecturer, 44 years).

“Student writing assessment has become unreliable. Many essays are not written by the students themselves.” (N8, Associate Professor, 45 years).

“This is my biggest anxiety as a teacher—discovering that AI-written content constitutes a high proportion of student submissions.” (N5, Associate Professor, 50 years).

Ethical anxiety compels teachers to reconsider fundamental pedagogical assumptions: What does it mean to assess learning when the boundary between student work and machine output is blurred? How can tasks be designed to elicit authentic student engagement? This anxiety thus becomes a catalyst for pedagogical innovation, pushing teachers to redesign assignments and recalibrate their relationships with students in the AI era.

### 4.3 The evolutionary pathway: three stages of adaptive transformation

Analysis of participants’ narratives revealed a patterned progression through three qualitatively distinct adaptive states. Importantly, progression is not automatic or unidirectional; teachers may oscillate between stages or become stuck at particular points depending on how their experiential wisdom is mobilized and the support available. Following Argyris and Schön’s (1974) distinction between “theories-in-use”and “espoused theories,” we classified stage membership based primarily on demonstrated behavioral evidence of actual classroom practices as reported in participants’ detailed narratives, with self-rated proficiency scores serving as supplementary contextual information rather than as definitive classification criteria. For each stage, we established explicit criteria grounded in (a) concrete descriptions of current, implemented teaching strategies and role redefinitions, and (b) participants’ self-rated proficiency as additional reference.

#### 4.3.1 Stage 1: exploration-adjustment

The interviewed teachers in this stage are characterized by (a) behavioral evidence of basic operational learning, coping with mandatory requirements, and initial critical scrutiny in their current practice, and (b) self-rated proficiency at the beginner level (1–2 out of 5) as a supplementary indicator. In the initial stage, teachers respond to technology anxiety by engaging in exploratory learning and basic skill acquisition. Their relationship with AI is characterized by cautious experimentation, often focused on mastering fundamental operations and understanding what the technology can do.

“If you’re asking about AI technology and platforms, I’d rate myself 2 points. I’m still a beginner, only using basic functions. I usually start by exploring on my own, then turn to online searches if I encounter problems.” (N2, Associate Professor, 48 years).

“I have barely engaged with it. Self-assessment: one point.” (N6, Associate Professor, 42 years).

At this stage, teachers begin to exercise critical scrutiny, an early expression of their experiential wisdom. Rather than accepting AI outputs uncritically, they compare them with their own knowledge and judgment. However, their critical engagement remains at the level of individual task completion, without systematic pedagogical redesign.

The exploration-adjustment stage represents teachers’ initial efforts to re-establish a sense of agency in the face of technological disruption. By actively seeking knowledge and critically evaluating AI outputs, they begin to position themselves as discerning users rather than passive recipients of technological change.

For some teachers, however, the exploration-adjustment stage represents a protracted state of stasis rather than a transitional phase. N2, an associate professor aged 48 with 23 years of experience, remained in this stage throughout her teaching career despite repeated efforts to progress: “I’ve tried many times to learn new AI tools, but I always revert to my old methods. … I’ve made peace with being a beginner.” Her self-rated proficiency (2 out of 5), admission of being a beginner, and description of reverting to established routines all indicate a pattern of cognitive entrenchment that institutional training alone could not overcome. Her case illustrates how experiential wisdom, when activated defensively as thinking set inertia, can anchor teachers in the exploration stage regardless of available support (Dane, 2010).

#### 4.3.2 Stage 2: strategy integration

The interviewed teachers in this stage are characterized by (a) behavioral evidence of consciously implemented pedagogical strategies (e.g., task redesign, keyword tactics, transforming AI limitations into teaching opportunities) to address AI-induced challenges, and (b) self-rated proficiency at the intermediate level (3 out of 5) as a supplementary indicator. As teachers gain confidence and competence, their focus shifts from “how to use” to “how to use well.” In this stage, technology becomes tactically integrated into pedagogical frameworks, often in response to problems created by technology itself, particularly students’ inappropriate AI use.

“AI can enrich my choices. I will filter and integrate based on my own teaching preferences, the actual situation of the students, and the overall course arrangement.” (N3, Lecturer, 43 years).

In this stage, AI transitions from being an “external intruder” to an “enabling tool.” Teachers’ experiential wisdom is mobilized to redesign tasks, redefine assessment criteria, and create learning opportunities from the challenges AI presents. Their subjectivity is strengthened as they demonstrate that professional judgment, not mere technical proficiency, is essential for meaningful technology integration.

However, progression to this stage is not always sustained. N5, an associate professor aged 50 with 27 years of experience, had developed innovative task designs to address students’ AI misuse. When her institution introduced new platform requirements, she experienced what she described as a regression: “Just when I thought I had figured out how to manage AI in my classroom, a new platform comes along and I have to start all over again.” Her trajectory illustrates how institutional change can disrupt even well-established adaptations, prompting temporary regression to earlier stages—underscoring the context-dependent nature of professional learning (Day and Gu, 2010).

#### 4.3.3 Stage 3: autonomous symbiosis

The interviewed teachers in this stage are characterized by strong and consistent behavioral evidence of pedagogical sovereignty and selective AI delegation in their current practice. Specifically, this includes: (1) selective delegation, where teachers demonstrate clear professional judgment in deciding which tasks are delegated to AI and which are retained for themselves; (2) pedagogical sovereignty in practice, evidenced by the active and creative modification, rejection, or critical repurposing of AI-generated content to fit specific pedagogical goals and student needs; and (3) proactive role redefinition, operationalized through the design of AI-resistant, complex tasks and the deliberate cultivation of humanistic educational elements.

Self-reported proficiency scores, which in our sample ranged from 3–5 out of 5, served only as additional contextual background and were not used as a threshold criterion. For instance, two participants (N7 and N10) with scores of 3 were classified in this stage because their detailed accounts of implemented strategies provided compelling evidence of all three behavioral hallmarks, consistent with our stage-classification criteria that prioritize theories-in-use over espoused beliefs. Teachers who reach the autonomous symbiosis stage have developed a confident, harmonious relationship with AI. Technology is no longer experienced as a threat but as a collaborative partner that handles routine tasks, freeing teachers to focus on the irreducibly human dimensions of education: emotional connection, value guidance, and fostering critical thinking. Cumulative mastery and peer validation served as triggers enabled teachers to move from tactical integration to autonomous symbiosis, confidently asserting their professional sovereignty in the AI era.

“The decision of whether to use AI, and how to use it, should be made by the teacher. We are the ones who understand our students and our teaching contexts.” (N9, Associate Professor, 59 years).

At this stage, teachers have achieved what might be termed pedagogical sovereignty in the digital age. They selectively deploy AI where it adds value, while confidently asserting their irreplaceable role in fostering students’ holistic development. Their professional identity is reconstructed around distinctly human strengths, and technology is experienced not as diminishing but as amplifying their capacity to educate.

### 4.4 The double-edged effects of experiential wisdom

A pivotal finding of this study is that mid-career teachers’ accumulated experiential wisdom functions as a double-edged sword which can either fuel evolutionary progress or entrench resistance, depending on how it is activated in the face of technological change.

#### 4.4.1 Experiential wisdom as evolutionary resource

When mobilized productively, experiential wisdom equips mid-career teachers with distinctive capabilities that younger teachers may lack. Three interrelated dimensions of wisdom-as-resource emerged from the data.

##### 4.4.1.1 Critical scrutiny capacity

Mid-career teachers draw on decades of disciplinary engagement to evaluate AI outputs with a discerning eye. They are not easily impressed by AI fluency; instead, they interrogate its content for accuracy, bias, and pedagogical appropriateness.

“Relying on our independent thinking abilities and long-term disciplinary accumulation, we can identify problematic stances and angles in AI-generated content. This is something less experienced teachers cannot do as readily” (N9, Associate Professor, 59 years).

“The first thing I do is verify the accuracy of AI-provided content. If I have doubts about a translation, I consult dictionaries and relevant documents to verify. You cannot simply trust AI.” (N3, Lecturer, 43 years).

##### 4.4.1.2 Pedagogical design wisdom

Teachers with extensive classroom experience possess a rich repertoire of pedagogical strategies. When confronted with AI, they creatively adapt these strategies to harness the technology’s potential while mitigating its risks.

“After teaching for over a decade, I know my students’ learning patterns well. I understand where they struggle. For example, pronunciation of certain sounds used to be difficult. Now I use AI to provide models and corrective feedback, integrating it seamlessly into my existing teaching framework.” (N1, Lecturer, 44 years).

“A colleague of mine collects student essays from before the AI era and compares them with AI-generated texts. She has students analyze the differences in emotional expression and personal voice. This turns AI from a threat into a tool for developing critical awareness.” (N8, Associate Professor, 45 years).

##### 4.4.1.3 Humanistic value commitment

Mid-career teachers consistently emphasize the irreplaceable human dimensions of education emotional connection, moral guidance, and the cultivation of thinking independently. Their experiential wisdom has taught them that teaching is fundamentally a relational practice, and they draw on this understanding to anchor their professional identity in an AI-mediated world.

“In areas like emotional understanding, thought guidance, and cultivating innovative perspectives, mid-career teachers have distinct advantages. We can provide feedback tailored to each student’s personality that AI cannot do.” (N3, Lecturer, 43 years).

“I consciously create opportunities for human connection in my classroom. For example, when students enter, I have them greet each other, chat briefly, which is a way to maintain the interpersonal warmth that online platforms cannot replicate.” (N1, Lecturer, 44 years)

#### 4.4.2 Experiential wisdom as evolutionary barrier

However, the same experiential wisdom that enables creative adaptation can also manifest as thinking set inertia, a cognitive and behavioral rigidity, which impedes change. Teachers whose professional identities are deeply invested in established routines may experience AI integration as a threat to their competence and self-worth.

“As age and teaching experience increase, thinking set becomes entrenched. There’s reluctance to change. Teaching methods, teaching rhythm, interaction patterns formed over many years become relatively fixed.” (N2, Associate Professor, 48 years).

“We’re accustomed to our the original teaching mode and not very willing to accept new things. It’s comfortable, and we know it works.” (N4, Professor, 43 years).

The effort to integrate AI while preserving established teaching styles creates significant cognitive and emotional strain.

“I want to maintain my original teaching style while incorporating AI, but it’s difficult and time-consuming. Finding the right balance requires much more effort than I anticipated.” (N2, Associate Professor, 48 years).

This struggle reflects the existential predicament of mid-career teachers: they must change to remain relevant, yet change threatens the very expertise that defines their professional identity. The resolution of this tension depends critically on whether experiential wisdom is reflexively examined and creatively repurposed, or defensively clung to as a bulwark against uncertainty.

### 4.5 Support systems enabling symbiotic adaptation

Teachers do not navigate the journey from anxiety to symbiosis in isolation. Analysis revealed four interconnected support systems that moderate the adaptation process, either facilitating progression or, when absent or misaligned, exacerbating difficulties.

#### 4.5.1 Differentiated professional development

Participants consistently criticized “one-size-fits-all” training programs that fail to address the specific needs and starting points of mid-career learners. They called for differentiated, practice-oriented professional development.

“I hope the university or education authorities can provide more targeted training. For example, Off the Job Training based on teachers’ current AI proficiency levels, rather than general sessions.” (N10, Associate Professor, 44 years).

“Current training lacks differentiation and practical components. Most are theoretical and not very effective. I would like future training to include more hands-on practice and real teaching cases.” (N3, Lecturer, 43 years).

“Institutions should not subjectively judge our learning needs; they should ask us what specific skills we want to develop.” (N9, Associate Professor, 59 years).

Effective training, from participants’ perspective, respects their prior knowledge while providing concrete, immediately applicable strategies for integrating AI into their specific teaching contexts.

#### 4.5.2 Collegial networks and cross-generational learning

Informal collegial networks within departments, across disciplines, and even extending to former students play a crucial role in supporting technology adaptation. These networks provide not only technical assistance but also emotional reassurance and validation.

“In our teaching and research section, there are colleagues who are more technologically proficient. I often consult them.” (N1, Lecturer, 44 years).

“Currently, I have no expert guidance. I explore together with my students. They comprehend faster than I do, and after they learn, they teach me. It’s reciprocal.” (N5, Associate Professor, 50 years).

“I maintain contact with graduated students; sometimes I ask them about new AI tools they use in their workplaces.” (N3, Lecturer, 43 years).

These intergenerational context learning communities exemplify what Wenger (1998) terms “communities of practice” that enable knowledge sharing and identity development. For mid-career teachers, such communities mitigate the isolation that can accompany technological change and provide affirming feedback that their experiential wisdom remains valued.

#### 4.5.3 Institutional recognition and evaluation reform

Participants emphasized that institutional evaluation systems must evolve to recognize the qualitatively different contributions teachers make in AI-enriched environments. Current systems that overemphasize technology usage metrics inadvertently undermine teachers’ sense of agency and value.

“Institutional policies should not excessively promote AI application at the expense of teachers’ predominance. For example, in teaching competitions, the role of AI should not be exaggerated in evaluation criteria.” (N9, Associate Professor, 59 years).

“I hope the university will improve relevant systems to comprehensively assess teaching ability and educational outcomes, including our contributions to student emotional development and critical thinking, not just technology adoption rates.” (N10, Associate Professor, 44 years).

Recognition reform, from participants’ perspective, would signal institutional validation of the humanistic dimensions of teaching that AI cannot replicate, thereby reinforcing teachers’ professional identity in the digital age.

#### 4.5.4 Resource adequacy

Finally, participants noted the importance of adequate resources, such as equipment, software access, protected time for learning, and technical support. Without these foundational enablers, even highly motivated teachers struggle to progress.

“We need reliable equipment and software, but also time. Learning AI while maintaining full teaching loads is exhausting.” (N6, Associate Professor, 42 years).

“Technical support should be readily available when we encounter problems. If we have to wait days for solutions, we lose momentum.” (N7, Professor, 48 years).

Beyond these formal support structures, institutional characteristics, specifically policy intensity, evaluation criteria, and resource availability, also appeared to shape teachers’ adaptation pathways. Teachers from institutions with strong top-down mandates for AI adoption exhibited more pronounced autonomy anxiety and were more likely to describe their relationship with technology in terms of compliance rather than voluntary adoption. Resource availability also emerged as a factor.

### 4.6 Summary

The findings reveal that mid-career foreign language teachers’ transition from technology anxiety to technology symbiosis is a complex, nonlinear process mediated by the activation mode of their experiential wisdom. Technology anxiety, in its four manifestations, provides the initial motivational energy. Teachers’ subsequent progression through exploration, integration, and symbiosis stages is shaped by whether their experiential wisdom is mobilized as a resource (critical scrutiny, pedagogical creativity, humanistic commitment) or remains trapped as inertia (thinking set rigidity). Support systems which is differentiated training, collegial networks, recognition reform, and resources moderate this process, either amplifying or dampening teachers’ capacity for adaptive transformation. The outcome, for those who navigate successfully, is a reconstructed professional subjectivity wherein teachers confidently assert their irreplaceable role as “learning designers” and “humanistic leaders” in AI-enriched educational environments.

## 5 Discussion

This study investigated how mid-career foreign language teachers navigate the transition from technology anxiety to technology symbiosis, focusing on the role of experiential wisdom in shaping adaptive trajectories. Below we discuss theoretical implications, engage with relevant literature, and consider practical applications for supporting this population.

### 5.1 Theoretical contributions: re-conceptualizing teacher subjectivity in the AI era

#### 5.1.1 Experiential wisdom as the pivotal mechanism

The most significant theoretical contribution of this study lies in identifying experiential wisdom’s dual effects as the pivotal mediating mechanism in mid-career teachers’ adaptation to AI. While previous research has acknowledged that experienced teachers possess resources for technology integration (Ertmer and Ottenbreit-Leftwich, 2010; Koehler and Mishra, 2009), the literature has largely treated experience as uniformly beneficial or, conversely, has framed teachers through deficit narratives (e.g., “digital immigrants”). Our findings complicate this picture by demonstrating that the same experiential repertoire can function as either an evolutionary resource or an evolutionary barrier, depending on how it is mobilized in response to technological disruption.

This duality advances existing theory in three ways. First, it challenges the implicit assumption in technology acceptance models (Davis, 1989) that usefulness perceptions are stable individual attributes^1^. Our findings suggest that for experienced teachers, perceptions of AI’s usefulness are filtered through existential concerns about professional identity preservation that TAM does not accommodate. Second, it extends research on expertise and adaptation by specifying when expertise facilitates versus impedes change. Cognitive psychology has documented that experts may develop cognitive entrenchment under certain conditions (Dane, 2010); our study identifies the specific conditions under which this occurs in educational settings, namely, when experiential wisdom is activated defensively as a protective response to identity threat. Third, it offers an identity-level explanation for why some mid-career teachers successfully adapt while others struggle: the critical variable is not the amount of experience per se, but the reflexive mobilization of that experience.

Our findings demonstrate that experiential wisdom is a particularly salient mechanism for mid-career teachers, whose professional identities have been forged over decades of practice and are therefore more deeply entangled with established routines than those of early-career teachers. Whether this mechanism operates similarly or differently across career stages remains an empirical question for future comparative research.

#### 5.1.2 The three-stage pathway: from technological disruption to pedagogical sovereignty

The identification of three progressive adaptive states, from exploration-adjustment, strategy integration, to autonomous symbiosis, which extends existing stage models of teacher technology adoption. While earlier models (e.g., the Concerns-Based Adoption Model; Hall and Hord, 2015) describe stages of concern and usage, they focus primarily on the technology itself and treat teachers as relatively undifferentiated. Our findings, by contrast, reveal a pathway organized around the reconstruction of professional subjectivity in which teachers progressively reclaim agency, redefine their pedagogical role, and achieve a qualitatively new relationship with technology^2^.

The exploration-adjustment stage corresponds to what Zhao et al. (2022) describe as the initial encounter with AI, characterized by technological shock and basic skill acquisition. However, our analysis reveals that even at this early stage, mid-career teachers begin exercising critical scrutiny, a distinctive manifestation of their experiential wisdom. This finding suggests that the adaptation process for experienced teachers is never purely technical; from the outset, it is infused with professional judgment and identity considerations.

The strategy integration stage represents a qualitative shift in teachers’ relationship with technology. At this point, teachers move beyond learning about AI to actively shaping how AI functions within their pedagogical ecosystems. The strategies they develop, such as task redesign, keyword tactics, transforming AI limitations into teaching opportunities, which exemplify what Tzafilkou et al. (2023) term pedagogical design capacity in technology-rich environments. Importantly, these strategies emerge in response to problems created by AI itself (particularly student misuse), illustrating the recursive relationship between technological change and pedagogical innovation.

The autonomous symbiosis stage represents the culmination of subjectivity reconstruction. Teachers at this stage have achieved what Biesta (2017) might call “pedagogical sovereignty,” the capacity to exercise professional judgment about when, how, and why to deploy technology, rather than having these decisions dictated by technological imperatives or institutional mandates. Their self-description as “learning designers” and “humanistic leaders” reflects a reconstructed professional identity in which technology is experienced not as diminishing but as amplifying their distinctively human contributions to education. This finding resonates with emerging international scholarship on teacher-technology symbiosis (Fang, 2025; Du and Ruan, 2025), while providing empirical specificity about how such symbiosis is achieved.

#### 5.1.3 The multidimensional nature of technology anxiety

Our identification of four distinct anxiety types: substitution anxiety, autonomy anxiety, generational anxiety, and ethical anxiety, which extends existing conceptualizations of teacher technology anxiety in several ways. Previous research has typically treated AI anxiety as a unitary construct or has distinguished only between cognitive, affective, and behavioral dimensions (Wang et al., 2022). Our findings reveal that mid-career teachers’ anxiety is existentially differentiated, showing different aspects of their professional being are threatened by different features of AI integration.

Substitution anxiety corresponds to what Selwyn (2022) describes as concerns about professional displacement, but our findings specify how this anxiety is experienced by language teachers confronting AI capabilities in their core disciplinary domains. The visceral reaction of N1, “Do we still need English majors?” The confusion reveals that substitution anxiety is not merely about job security but about the meaning and value of a professional life devoted to language teaching.

Autonomy anxiety resonates with international concerns about teacher deprofessionalization under technological rationalization (Ball, 2016; Biesta, 2017). However, our findings highlight a distinctive feature of the Chinese higher education context: institutional mandates for technology use, often embedded in platform architectures and evaluation systems, create conditions where teachers experience technology as imposed rather than chosen. This institutional dimension of anxiety has received less attention in Western literature, which tends to focus on individual psychological factors.

Generational anxiety reflects the specific predicament of mid-career teachers positioned between digital native students and younger colleagues. While digital immigrant narratives have been critiqued for oversimplifying generational differences, our participants’ accounts suggest that the experience of generational comparison is psychologically real and consequential. This anxiety is intensified in the Chinese cultural context, where seniority traditionally confers authority and respect. When senior teachers find themselves less technologically fluent than their juniors, the resulting identity threat can be profound.

Ethical anxiety emerges from the disruption of fundamental pedagogical relationships and assumptions. Teachers’ concerns about academic integrity, student engagement, and the meaning of assessment in an AI-saturated world reflect what Zhao et al. (2024) term the “existential crisis” underlying technology anxiety. Notably, ethical anxiety, while distressing, also proved generative which was precisely the breakdown of traditional teaching assumptions that motivated many teachers to redesign tasks and reimagine their pedagogical roles.

The theoretical implication is that effective support for mid-career teachers must address each anxiety type distinctly. Skill training may alleviate some aspects of generational anxiety but will not resolve substitution or ethical anxieties, which require existential and pedagogical reflection. Institutional policies that mandate technology use may inadvertently exacerbate autonomy anxiety unless accompanied by opportunities for genuine teacher input and adaptation.

#### 5.1.4 The moderating role of support systems

Our findings confirm and extend research on the importance of support systems for teacher technology integration (Liu et al., 2017). However, our analysis reveals that support is the most effective when it is differentiated according to teachers’ adaptive stage and aligned with their specific anxiety sources.

The call for differentiated professional development resonates with international practices in teacher learning (Opfer and Pedder, 2011), yet our findings suggest that differentiation must consider not only teachers’ current skill levels but also their stage in the subjectivity evolution process. Teachers in the exploration stage need basic skill training combined with reassurance that their experiential wisdom remains valuable. Those in the integration stage benefit from opportunities to share and refine pedagogical strategies with peers facing similar challenges. Teachers approaching symbiosis require platforms to articulate and disseminate their reconstructed professional identities.

The importance of collegial networks and cross-generational learning communities echoes research on communities of practice (Wenger, 1998) and teacher collaboration (Vangrieken et al., 2015). Our findings add specificity by revealing how such communities function for mid-career teachers, providing not only technical assistance but emotional validation and identity affirmation. The reciprocal learning relationships some participants developed with students (N5: “they learn and then teach me”) are particularly significant, as they transform the generational hierarchy that generates anxiety into a resource for mutual growth.

The call for evaluation reform addresses a critical but often neglected dimension of teacher support. When institutional evaluation systems emphasize quantifiable technology usage metrics, they inadvertently signal that technology adoption is an end in itself, undermining teachers’ sense of pedagogical agency. Participants’ desire for recognition of their humanistic contributions, such as emotional connection, value guidance, critical thinking cultivation, reflects a sophisticated understanding of what makes teaching irreplaceable in the AI era. Institutionalizing such recognition would provide powerful validation for the professional identities teachers are reconstructing.

### 5.2 Connecting to international research on mid-career teachers

International research on mid-career teachers has emphasized the “career plateau” phenomenon—stabilized routines, reduced promotion prospects, and potential for either renewal or stagnation (Hargreaves and Fullan, 2012; Ismail, 2008). Comparative international studies have further illuminated the nuanced realities of this career phase. Shin et al. (2023) interview study with 16 mid-career foreign language teachers across Austria and England found that these teachers benefitted from an established sense of confidence but also actively sought further professional growth opportunities, drawing positivity from the meaning they took from their work while occasionally experiencing tensions between job demands and wellbeing. Our findings suggest that AI disruption creates both risks and opportunities at this career stage. The risk is that technology anxiety compounds plateau-related dissatisfaction, pushing teachers toward disengagement, whereas the opportunity is that successful navigation of AI integration can catalyze professional renewal, enabling teachers to reconstruct their professional identity around new sources of meaning. This resonates with Day and Gu’s (2010) research on teacher resilience, which emphasizes that sustaining commitment depends on finding renewed purpose across career phases. For the teachers in our study, AI integration, despite its anxieties, offered precisely such an opportunity for renewal.

### 5.3 Practical implications

#### 5.3.1 Designing stage-sensitive professional development

Our findings suggest that professional development for mid-career teachers should be calibrated to their position in the subjectivity evolution process:

For teachers in the exploration-adjustment stage, professional development should combine basic skill training with psychological reassurance. Programs should acknowledge the legitimacy of anxiety while providing concrete, immediately applicable strategies. Most importantly, they should affirm that teachers’ existing expertise remains valuable and learning technology does not require abandoning hard-won pedagogical wisdom.

For teachers in the strategy integration stage, professional development should create opportunities for sharing and refining pedagogical strategies. Problem-based workshops focused on real classroom challenges (e.g., redesigning assignments to maintain academic integrity) enable teachers to pool their collective wisdom and learn from peers facing similar dilemmas. Cross-institutional networks can amplify these benefits by exposing teachers to diverse approaches.

For teachers approaching autonomous symbiosis, professional development should provide platforms for articulating and disseminating reconstructed professional identities. Mentoring programs that pair these teachers with colleagues earlier in the adaptation process can simultaneously validate their expertise and support others’ growth. Opportunities to contribute to institutional policy discussions about technology integration can reinforce their sense of agency and influence.

#### 5.3.2 Fostering supportive institutional cultures

Beyond formal professional development, institutions can cultivate conditions that support mid-career teachers’ adaptation:

1 Recognition reform: Evaluation systems should explicitly value the humanistic dimensions of teaching that AI cannot replicate, such as emotional connection, value guidance, critical thinking cultivation. This signals institutional validation of the professional identities teachers are reconstructing.
2 Cross-generational learning structures: Institutions should intentionally create opportunities for reciprocal learning between mid-career and early-career teachers, as well as between teachers and students. Such structures transform generational differences from sources of anxiety into resources for collective growth.
3 Protected time and resources: Learning AI while maintaining full teaching loads is exhausting. Institutions should provide dedicated time for exploration and collaboration, along with reliable technical infrastructure and support.
4 Autonomy-preserving policies: While institutional technology initiatives are inevitable, they should preserve space for teacher judgment and adaptation. Mandates that prescribe specific tools or usage patterns should be accompanied by opportunities for teacher input and modification.

#### 5.3.3 Implications for teacher education

Our findings also hold implications for initial teacher education. If mid-career teachers’ adaptation challenges center on the reflexive mobilization of experiential wisdom, then pre-service preparation should cultivate capacities for adaptive expertise to recognize when established routines require modification and to creatively repurpose existing knowledge for novel situations (Hatano and Inagaki, 1986). Rather than merely transmitting current best practices, teacher education should prepare novices for a career of continuous learning and identity reconstruction in response to technological and social change.

### 5.4 Limitations and future research directions

Several limitations should be acknowledged. First, the study’s sample is geographically concentrated in Guangxi (60%), with additional participants from Guangdong, Jilin, and Fujian provinces. While maximum variation sampling was employed to ensure diversity along theoretically relevant dimensions, namely age, teaching experience, academic rank, institutional type, and self-rated technology competence, rather than geographic representativeness, we acknowledge that this geographic imbalance limits the transferability of our findings to other regional contexts. The distribution of institution types is also unbalanced. Science and technology universities are overrepresented, while foreign studies universities, normal universities and comprehensive universities are underrepresented. This imbalance reflects the practical constraints of our recruitment channels rather than a deliberate sampling design. Comparative studies across different regional, educational, and disciplinary settings are needed to examine how macro-level factors shape subjectivity evolution.

Second, the study relies on retrospective self-report data, which carries inherent limitations, including recall bias and post-hoc rationalization. The three-stage model should therefore be understood as a representation of participants’ meaning-making and narrative reconstruction of their experiences, rather than an objective timeline verified through longitudinal tracking. This orientation is consistent with our constructivist grounded theory approach (Charmaz, 2014), which treats participants’ interpretations as valid data for theorizing subjective experience. Future research employs longitudinal designs, such as diary studies or experience sampling, which could complement our findings by capturing real-time dynamics.

Finally, AI technology evolves rapidly. While specific tools triggering anxiety will be superseded, the existential dynamics we identified the relationship between technological change, experiential wisdom, and identity reconstruction that may prove more durable. Longitudinal research tracking cohorts across successive technological transitions could test the model’s robustness over time.

## 6 Conclusion

This study employed constructivist grounded theory to investigate how mid-career foreign language teachers in Chinese universities navigate the transition from technology anxiety to technology symbiosis in the era of generative AI. Through in-depth interviews with ten purposefully selected teachers aged 40 and above, we developed a theoretical model of mid-career foreign language teachers’ subjectivity evolution mechanism. There are three theoretical contributions warrant emphasis.

First, the identification of experiential wisdom as a double-edged mechanism challenges deficit-oriented narratives about older teachers, revealing that the same expertise enabling sophisticated judgment can also engender cognitive entrenchment when defensively mobilized.

Second, the multidimensional conceptualization of technology anxiety extends existing psychological models by revealing the existential sources of teacher distress that skill training alone cannot resolve.

Third, the three-stage model of subjectivity evolution provides a processual understanding of how teachers reconstruct professional identity through engagement with technology, offering practical guidance for stage-sensitive intervention design.

The integration of AI into foreign language education is a permanent transformation. For mid-career teachers, this presents both challenges and opportunities to re-examine assumptions and reconstruct professional identity around distinctively human strengths. The teachers in this study confound stereotypes of older educators as technologically resistant. Their narratives reveal creativity, agency, and resilience in navigating disruption. Successful adaptation is not about becoming “more like” younger teachers, but about drawing upon experiential resources to forge new professional identities suited to the AI era.

For researchers, this study invites continued attention to the existential dimensions of teacher-technology relationships. For practitioners and policymakers, it calls for support systems that help mid-career teachers mobilize experiential wisdom as a resource rather than allowing it to become a barrier.

As one participant expressed: “The decision of whether to use AI, and how to use it, should be made by the teacher. We are the ones who understand our students.” In this statement lies the essence of autonomous symbiosis: technology as tool, teacher as sovereign, education as fundamentally human.

## Data Availability

The original contributions presented in the study are included in the article/Supplementary material, further inquiries can be directed to the corresponding author/s.

## Footnotes

1 Traditional technology acceptance models (TAM) focus on perceived usefulness and ease of use as predictors of adoption (Davis, 1989), but cannot explain why the same individual may both embrace and resist technology depending on how their existing expertise is activated. Our findings suggest that for experienced teachers, usefulness perceptions are filtered through existential concerns about professional identity preservation in which TAM does not accommodate. Recent efforts to incorporate emotional factors into technology acceptance models (Wang et al., 2022) represent progress, but our study further specifies that experiential wisdom itself mediates how usefulness and ease of use are interpreted.

2 The TPACK framework (Koehler and Mishra, 2009) conceptualizes the knowledge teachers need for technology integration as an intersection of technological, pedagogical, and content knowledge. Our findings affirm this framework while suggesting an identity dimension: transforming rich pedagogical content knowledge into TPACK is not purely cognitive but requires professional self-reconstruction. Mid-career teachers in our study needed opportunities not just to acquire new knowledge, but to reflexively examine how technology integration aligned with or challenged their sense of who they are as teachers.
