# Supplementary material for: From technology anxiety to technology symbiosis: a grounded theory study of mid-career foreign language teachers’ subjectivity evolution in the AI era
Source: Front Psychol. 2026 Sep 17;17:1914637. doi: 10.3389/fpsyg.2026.1914637 (PMC13627022; doi:10.3389/fpsyg.2026.1914637)
Supplement: Supplementary file 1 [file Supplementary_file_1.docx]

**Appendix B: Semi-Structured Interview Protocol**

Introductory Script

Dear sir/madam,

We sincerely thank you for taking the time to participate in this interview. We are conducting a study to gain an in-depth understanding of university foreign language teachers’ authentic experiences, perceptions, strategies, challenges, and needs in interacting with AI technology in their teaching, with particular attention to how teachers achieve “symbiosis” with AI technology. This study focuses on foreign language teaching contexts. All interview content will be kept strictly confidential and used solely for academic research purposes. All identifying information will be anonymized in any publications, and there will be no adverse consequences for you personally. Please feel free to speak openly based on your actual teaching experience, and the more detailed your sharing, the better.

We sincerely appreciate your valuable time and candid sharing! We wish you all the best in your work and life!

Module 1: Background Information

Could you briefly introduce yourself: Your university, courses you teach, your age, years of teaching experience, and academic rank?

Module 2: Technology Experience and Perceptions

1.In your daily foreign language teaching, what AI technology tools or platforms do you primarily use? What benefits do they bring to your teaching and research?

2.How would you rate your current level of AI technology application proficiency? On a 5-point scale, what score would you give yourself?

3.When you need to learn a new AI technology, how do you typically go about learning it? (Probe: Do you explore independently, consult colleagues, attend training, or use other approaches?)

4.In the process of learning to use new technologies, what specific difficulties have you encountered? How did you try to resolve them? Please describe 1–2 specific learning experiences in detail.

5.Have any colleagues, students, or others provided you with particularly important technical assistance? How did they help you?

Module 3: Multidimensional Exploration of Technology Anxiety

6.When AI systems (e.g., intelligent essay scoring/translation practice evaluation, AI oral proficiency assessment, AI learning analytics such as vocabulary weakness diagnosis) take over core teaching tasks traditionally led by you (e.g., detailed grading, personalized feedback, diagnosing learning difficulties), have you ever felt that your professional value or irreplaceability has been diminished? Has this affected your career prospects?

7.When using AI-recommended teaching content (e.g., AI-generated reading materials, topics), activity designs (e.g., AI-suggested classroom games), or resources (e.g., AI-pushed grammar exercises), have you felt that your autonomy in curriculum design has been constrained? Has this affected your teaching decisions?

8.In the face of AI’s powerful capabilities in information retrieval (e.g., massive corpora), language knowledge explanation (e.g., complex grammar points), translation speed and breadth, speech imitation, and even intercultural knowledge Q&A, have you felt that your traditional authority in language knowledge, translation skills, or cultural background knowledge has been challenged?

9.Do you worry that over-reliance on technology may weaken your educational judgment or humanistic care? Does prolonged use of AI technology generate a sense of burnout in your work?

10.When technology (e.g., online teaching platforms, AI-mediated communication tools) becomes the primary channel for interaction with students, do you feel that the absence of non-verbal communication (facial expressions, body language, immediate classroom atmosphere) affects your understanding of students (e.g., whether they are confused, engaged, or their emotional states) and your empathy toward them?

11.When using AI tools (especially those involving students’ foreign language assignment data, AI-algorithm-recommended learning resources or pathways, AI automated evaluation results such as writing scores or oral proficiency levels), what are your primary concerns?

Module 4: Challenges, Support, and Needs

12.In terms of technology use, what do you perceive as the most significant unique challenges you face compared to younger foreign language teachers or digital-native students?

13.How do you draw on your extensive foreign language teaching experience and pedagogical wisdom to balance or counteract the potential negative impacts of AI?

14.When applying AI tools (e.g., translation engines, writing assistants, resource recommendation systems), how do you maintain critical scrutiny?

15.Has the existing support (training, policies, etc.) been effective in helping you cope with technology anxiety? What assistance has your institution provided to support mid-career foreign language teachers’ technology application? What more specific or targeted support would you like your institution or education authorities to provide to help you better utilize technology?

Module 5: Understanding and Vision of “Technology Symbiosis”

16.Based on your experience, how do you understand the “symbiotic” relationship between foreign language teachers and technology? How would you describe the current state of your relationship with technology in your foreign language teaching? (Harmonious collaboration? Barely adapting? Mutual conflict?)

17.In your ideal scenario (particularly after effectively addressing the anxieties mentioned above), what would the “symbiotic” relationship with AI technology look like? In your foreign language classroom, how would you envision the interaction among teachers, students, and technology? What role would technology play? How would the teacher’s core value be manifested?

18.To achieve this healthier and more sustainable symbiosis, what do you believe needs to change:

At the individual level (teacher mindset, skills): What key changes are needed?

At the external environment level (institutional systems, culture, support): What key changes are needed?

Closing Script

Thank you for your support and valuable sharing. Your insights are extremely helpful for our research. We will ensure that all your responses are kept confidential and anonymized in any publications. May we contact you again if we have any follow-up questions? Thank you again!
