# Supplementary material for: From technology anxiety to technology symbiosis: a grounded theory study of mid-career foreign language teachers’ subjectivity evolution in the AI era
Source: Front Psychol. 2026 Sep 17;17:1914637. doi: 10.3389/fpsyg.2026.1914637 (PMC13627022; doi:10.3389/fpsyg.2026.1914637)
Supplement: Supplementary file 2 [file Supplementary_file_2.docx]

| **25 Conceptualizations with Original Data Examples** | |
| --- | --- |
|  |  |
| Conceptualization | Original Data Examples (Respondent Number-Statement Number) |
| Technological Shock | I felt it was too powerful, and at that moment I was very anxious, wondering whether we would still need English majors or translation majors in the future. The pressure was immense. (N-1-27); "AI can automatically correct on the basis of understanding, which ability is very strong." (N-1-26); "I was shocked when I first encountered ERNIE Bot‌." (N-1-25) |
| Pedagogical pain points | My university English classes have very large enrollments... the enormous workload causes serious teaching difficulties.” (N-10-7); "Lesson preparation saves time and effort, and it solves the problem of insufficient class hours." (N-2-5); "When assigning large-scale writing assignments, it can automatically grade and score, which is highly efficient." (N-3-5); "The backend of the Learning Platform Chaoxin Learning can record students' learning processes. In an ideal scenario, I could use this data to complete paper writing or research work without having to manually process numbers." (N-2-5) |
| Mandatory Use Pressure | Some school management systems mandate the use of AI functions, such as attendance tracking, but I am accustomed to manual attendance-taking. I can only barely cope with such mandatory requirements. (N-9-21) |
| Evaluation System Pressure | In competition evaluations, we should not overemphasize the role of AI application outcomes in professional title assessments, so as to prevent teachers from seeking quick success and instant benefits. (N-9-22); "I hope the school can improve the relevant systems, for example, by establishing a more reasonable teacher evaluation system that does not overemphasize the outcomes of AI technology application." (N-10-19) |
| Student Dependency Behavior | When assigning role-play tasks, students used to think—even if they were just reading mechanically from the script. Now they directly input the task into AI and copy the generated dialogue, lacking any thinking process. (N-1-9); "I am concerned about students' over-reliance on AI, which leads to a decline in their autonomous learning and independent thinking abilities." (N-3-15); "Many students did not write the essays themselves. They may have written in Chinese first and then had AI translate it, but the platform cannot detect this." (N-8-19); "This is my greatest anxiety as a teacher. When I was teaching English writing as a substitute, I found that a very high proportion of students' writing was generated by AI." (N-5-12); "When assigning translation or composition homework, some students tend to use AI to complete it." (N-2-25); "The proportion of students using AI for writing is very high." (N-5-12) |
| Weakening of Interpersonal Communication | In the past two years since the advent of AI, I have felt that students are increasingly immersed in their own interactions with their phones, and interpersonal communication has diminished. (N-1-15); "Even before the emergence of generative AI, when using these platforms, I already felt that the teacher-student relationship was becoming increasingly distant." (N-8-17); "AI's responses are not human enough—they are formulaic, like customer service chatbots, and often provide irrelevant answers." (N-4-15); "Students will ask AI first, and only ask the teacher when they are uncertain." (N-4-15) |
| Shift of Knowledge Authority | I can clearly feel that students ask fewer questions—they all go to ask AI instead. (N-8-13); "In the past, teachers were superior to students in both the breadth and depth of knowledge. But now students have abundant channels to access information, and they have surpassed teachers in many areas based on their interest-driven in-depth learning—teachers' academic authority has been impacted." (N-9-14); "In teaching, I guide students to discern and filter the information provided by AI by considering context and cultural background... but the impact of AI cannot be ignored." (N-3-12) |
| Integration-stage relational anxiety | When AI assists in grading essays and processing translations, I feel more strongly that my professional value is being diminished. (N-2-10); "After comparing many translations, I have a strong sense of anxiety—AI translation is fast and accurate..." (N-5-8); "At that moment I was very anxious, wondering whether we would still need English majors or translation majors in the future—the pressure was immense." (N-1-27); "AI translation is fast and accurate, but it falls short of human translation in handling certain details." (N-5-8) |
| Loss of Teaching Order Control | This is my greatest anxiety as a teacher. When I was teaching English writing as a substitute, I found that the proportion of AI-generated writing was very high. (N-5-12); "Before assigning homework, I emphasize the importance of independent thinking, and during class, I strictly monitor students' use of electronic devices." (N-5-12) |
| Conflict-Resistance Stage | I have barely engaged with it, because I am currently pursuing a doctoral degree and have not really studied AI in terms of teaching. (N-6-5); "Self-assessment: one point." (N-6-6); "The acceptance of new technologies is relatively low—at first I resisted, feeling that there was no need to learn it." (N-3-16) |
| Exploration-stage characteristics | Does this include both AI technologies and platform usage? If so, I think I would give myself 2 points—I am still a beginner and can only use basic functions; I cannot say I am good at it. (N-2-6); "I think I haven't even passed—probably around 2 to 2.5 points." (N-3-6); "I am not yet proficient in using AI... for example, when I use AI to generate quiz questions, the questions are rather superficial, because I am not skilled at setting prompts." (N-5-9); "At most a passing score—2.5 to 3 points." (N-8-6) |
| Strategy-Integration Stage | AI can enrich my choices. I will filter and integrate based on my own teaching preferences, the actual situation of the students, and the overall course arrangement. (N-3-11); "I generally combine both approaches... if the prompts are well-designed, what AI provides is quite good—more comprehensive than what I could think of on my own—and I will selectively modify and adjust it." (N-1-13); "I will first determine my own teaching direction, and then use AI as a supplement." (N-4-12); "I usually make selective modifications and do not accept everything completely." (N-7-11); "There are considerable discrepancies... I must make selective modifications—AI's suggestions are sometimes too far-fetched." (N-8-12); "I do refer to AI, and to a large extent, because most of its suggestions are quite good." (N-5-9) |
| Autonomous-Symbiosis Stage | I believe it is a state of harmonious symbiosis. I have been making continuous efforts to learn AI technology and apply it in my teaching practice when appropriate. (N-7-20); "In the field of foreign language teaching and linguistic culture, the relationship between humans and AI is one of harmonious symbiosis." (N-9-21); "I think it should be considered a state of harmonious collaboration." (N-10-17); "At present, I am basically able to apply AI technology to all aspects of teaching—pre-class lesson preparation, in-class instruction, and after-class assignment monitoring." (N-10-17) |
| Experience as resource: Critical scrutiny | With our independent thinking ability and years of disciplinary accumulation, we can identify problems of stance and perspective. (N-9-13); "First, I need to verify the accuracy of the content provided by AI... when I have doubts about translation content, I verify it by consulting dictionaries and literature." (N-3-17); "AI merely filters from a vast pool of fed resources—these resources are not necessarily correct, and we need to control their accuracy and value." (N-4-12); "When using AI, one must maintain a critical stance—it has value, but it cannot be used without discernment." (N-4-14); "For translation tools, I focus on the cultural and artistic aspects of the translation results... for writing assistants, I focus on the relevance of the generated content to the topic and whether the viewpoints align with correct value orientations." (N-10-15) |
| Task design innovation | Having taught for over a decade, I know the students' learning situations and know how to use AI to compensate for deficiencies. (N-1-19); "Some teachers of my age compare articles written by students before the advent of AI with articles generated by AI, allowing students to experience the emotional differences—this approach demonstrates great wisdom." (N-8-21); "Teachers have advantages in emotional understanding, thought guidance, and viewpoint innovation. Teachers can provide feedback based on students' personality traits and offer guidance that is more tailored to students' actual circumstances based on teaching experience." (N-3-9); "I will make the tasks more complex... the tasks are very complicated, requiring students must read through all the information and integrate it according to the key points we provide in order to complete the task." (N-1-10); "When assigning paragraph translation homework, I give specific keywords that must be used—this way, they cannot meet the requirements by using translation software alone, so they use AI technology while also having to think for themselves." (N-2-25) |
| Adherence to Humanistic Values | Teachers have advantages in emotional understanding, thought guidance, and viewpoint innovation. (N-3-9); "Taking essay grading as an example, AI has deficiencies in emotional judgment. For the same essay, from the perspective of emotional expression, a teacher's score may be more than 10 points higher than AI's." (N-7-9); "Students may care more about the emotional support and value guidance provided by teachers in traditional classrooms." (N-2-17); "AI finds it difficult to understand the emotional expression in essays." (N-7-10); "The core value of teachers is embodied in the overall design of teaching, the guidance and education of students, and the transmission of correct values—these are all things that AI cannot replace." (N-10-18) |
| Experience as barrier: Thinking set | With the increase of age and teaching experience, there is a fixed mindset and an unwillingness to change. The teaching methods, styles, classroom pacing, and interaction patterns formed over many years are relatively rigid. (N-2-16); "We are accustomed to the original teaching model and are not willing to accept new things." (N-4-16); "The challenge lies in the lower speed and degree of acceptance of new things." (N-4-16); "Learning speed is slow, and I forget quickly if I don't use what I've learned." (N-3-16) |
| Sense of Powerlessness in Technology Integration | It is difficult to maintain my original style while integrating AI technology—it requires more time. (N-2-16); "The biggest challenge is that the materials generated by AI do not meet expectations... even if I keep changing the prompts, I may not get satisfactory results." (N-7-6); "The integration of AI into course design has not been done well." (N-8-11) |
| Colleague Support Network | I don't have a fixed person to consult, but sometimes I communicate with colleagues and ask each other when we encounter something we don't understand. (N-1-8); "Through communicating with different colleagues, I can learn something from each of them—they share their tips on using AI tools." (N-10-6); "When I was learning before, the newly recruited young colleagues were more familiar with the relevant technologies—I often consulted them, and they were very willing to help." (N-6-8); "A colleague introduced me to Doubao." (N-4-9); "I can learn a lot from young colleagues." (N-4-9); "In our teaching and research office, Youqi and Maojun are more forward-thinking, and I communicate with them." (N-1-8) |
| Teacher-Student Learning Community | There is currently no professional guidance—I explore together with the students. When encountering problems, I search on Xiaohongshu. The students have stronger comprehension abilities than I do, and they teach me after they learn. (N-5-7); "I will form a learning community with the students and grow together." (N-5-5); "Sometimes students know quite a lot, and I also ask them." (N-10-4); "Once, I used AI to generate copywriting and text-to-image with the students—it actually brought us closer together." (N-5-11) |
| Training: Need for precision | I hope the school or higher-level authorities can provide more targeted training—for example, tiered training based on teachers' different levels of AI technology application proficiency. (N-10-16); "If there were zero-baseline AI training, older teachers would feel less psychological burden... tiered training would be better." (N-1-22); “I prefer targeted training lasting about a week... for older teachers, a model combining training with practice works better.” (N-7-17); "Current training is organized by topic and cannot adjust the pace according to age." (N-1-22); "There is no need for categorization—older teachers can also master the knowledge if they persist in learning. However, the application phase could appropriately extend the time, and a model combining theory and practice would be better." (N-7-18) |
| Demand for Practical Training | The training lacks differentiation and practical hands-on components—most of it is too general and the results are not ideal. (N-3-18); "For older teachers, a model that combines training with simultaneous practice works best, to avoid having too much content to digest." (N-7-17); "The school does not offer age-stratified training—all training is conducted uniformly, in both online and offline formats... the results are not ideal." (N-3-18); "I can tell that the school puts a lot of effort into training and the quantity is large, but it's similar to large-class lectures—the results are not very good." (N-5-17) |
| Demand for Resources and Time Guarantees | The school needs to provide equipment and financial support... sharing experiences among colleagues is very important, but currently everyone is too busy and lacks the time and energy for exchange and sharing—this is a common problem faced by middle-aged teachers. (N-5-19); "The school needs to provide training funding—short-term lectures have limited effectiveness; teachers need financial support for in-depth learning." (N-9-19); "Many AI tools require purchasing memberships, which is costly for students to use." (N-5-6); "Due to funding constraints, I can only make videos myself, and the results are not as good as those produced by professional teams." (N-5-6); "Some tools can only be used abroad and cannot be accessed within China, and some require payment." (N-1-7) |
| Demand for Reasonable Evaluation Systems | I hope the school can improve the relevant systems, for example, by establishing a more reasonable teacher evaluation system that does not overemphasize the outcomes of AI technology application. (N-10-19); "We should not overemphasize the role of AI application outcomes in professional title assessments." (N-9-22); "The teaching evaluation system should also be adjusted with the development of AI technology, clarifying the proportion of process and results in the evaluation." (N-2-24); "We should not neglect the dominant position of teachers by overemphasizing AI application." (N-9-22) |
| Active Learning Exploration | I am accustomed to exploring on my own first. If I cannot figure it out, I search online for solutions to further master the technology. (N-2-7); "Mainly self-study... there are many online learning resources, such as Bilibili videos and Baidu searches, to understand the general操作方法." (N-4-7); "Mainly through paid training—I do relatively less self-exploration." (N-9-9); "I usually explore on my own first... I also consult colleagues, and sometimes students know quite a lot as well." (N-10-4); "I followed the school's training to learn... and then tried to use it on my own." (N-1-6); "I sign up for any training opportunities that arise. Beyond that, I also explore on my own." (N-7-4); "There are two main ways I learn AI technology—one is participating in training organized by the school, and the other is exploring in practice." (N-5-5) |
